# Indoor Solar Thermal Energy Saving Time with Phase Change Material in a Horizontal Shell and Finned-Tube Heat Exchanger

**DOI:** 10.1155/2015/291657

**Published:** 2015-03-23

**Authors:** S. Paria, A. A. D. Sarhan, M. S. Goodarzi, S. Baradaran, B. Rahmanian, H. Yarmand, M. A. Alavi, S. N. Kazi, H. S. C. Metselaar

**Affiliations:** ^1^Department of Mechanical Engineering, Faculty of Engineering, University of Malaya, 50603 Kuala Lumpur, Malaysia; ^2^Department of Mechanical Engineering, Faculty of Engineering, Assiut University, Assiut 71516, Egypt; ^3^Department of Mechanical Engineering, Mashhad Branch, Islamic Azad University, Mashhad, Iran; ^4^Department of Mechanical Engineering, Jahrom Branch, Payame Noor University, Jahrom, Iran

## Abstract

An experimental as well as numerical investigation was conducted on the melting/solidification processes of a stationary phase change material (PCM) in a shell around a finned-tube heat exchanger system. The PCM was stored in the horizontal annular space between a shell and finned-tube where distilled water was employed as the heat transfer fluid (HTF). The focus of this study was on the behavior of PCM for storage (charging or melting) and removal (discharging or solidification), as well as the effect of flow rate on the charged and discharged solar thermal energy. The impact of the Reynolds number was determined and the results were compared with each other to reveal the changes in amount of stored thermal energy with the variation of heat transfer fluid flow rates. The results showed that, by increasing the Reynolds number from 1000 to 2000, the total melting time decreases by 58%. The process of solidification also will speed up with increasing Reynolds number in the discharging process. The results also indicated that the fluctuation of gradient temperature decreased and became smooth with increasing Reynolds number. As a result, by increasing the Reynolds number in the charging process, the theoretical efficiency rises.

## 1. Introduction

The general interest in renewable energy source applications, particularly solar energy, has increased as a result of paying closer attention and extended research on environmental considerations along with effective and rational energy management. Due to disparities between solar heating energy supply and demand, employing devices to store thermal energy is necessary. Thermal energy storage plays a significant role in practical and rational energy usage, since the dominance of energy demand over production is globally recognized. Suitable thermal storage is particularly necessary in cases where intermittent energy is generated, such as waste heat recovery and solar thermal systems. Energy storage can be defined as the storage of a form of energy which can later be utilized in other advantageous operations. Thermal energy storage can refer to technologies through which energy is stored in a thermal reservoir for later purposeful utilization. It is also possible to maintain the thermal reservoir at a lower (colder) or higher (hotter) temperature than that of the ambient environment.

There are three main ways of storing thermal energy, namely, sensible, latent, and thermochemical heat storage. However, the mismatched energy availability and demand for solar domestic hot water (SDHW) systems as well as limited availability of solar energy at night are problems that need to be addressed [1]. Thermal energy storage (TES) is commonly used in bridging the gap between energy demand and availability. Hot water storage tank systems are required to store sensible energy; however, they need significant space and add weight to structural components. Such requirements inhibit further SDHW deployment in buildings with limited space or structural limitations. Nevertheless, using phase change materials (PCMs) for TES can solve this problem by reducing the weight and space required for energy storage [2]. Thermal storage technology based on using PCM has attracted increasing attention in recent years. This is mostly attributed to the high density of energy storage occurring in the process of phase change within a very narrow temperature range. They can be used in balancing the night and day-time energy demand. PCMs function as energy storage mediums, whereby energy is stored during the melting process and is released during solidification. PCMs have the advantage of having isothermal operating attributes, such as heat charging and discharging at an almost constant temperature along with high energy storage density during melting and solidification. These benefits make PCMs an appropriate choice for effective thermal system operation [3].

Recently, several works have been carried out to study the thermal characteristics of paraffin during the melting and solidification processes [4–10]. Paraffin wax PCMs were found to have good thermal stability after repeated cycles using a DSC, showing little to no degradation of the latent heat and phase transition temperature ranges [11, 12]. Dodecanoic acid (also called lauric acid) has been shown to have a melting temperature range that is suitable for LHESS used for SDHW energy storage (40 to 50°C for a SDHW system) [13]. It also has stable thermal properties, is safe for use with domestic water, and is readily available and relatively inexpensive [14]. Two methods of using PCM to store thermal energy can be found in the literature. One of the methods involves using PCM as a wax in the shell and tube heat exchanger. In this type of heat storage unit, the PCM is placed in the shell and the HTF flows through the tubes. A number of researches have been conducted on this configuration by Zhang and Faghri [15], Lacroix [16], Cao and Faghri [17], Ismail and Lino [18], Akgün et al. [19], Liu et al. [20, 21], and Bellecci and Conti [22]. The rigid capsule is the second configuration. In this method, PCM is placed in the capsule and HTF flows through a tube surrounding the capsule.

The shell and tube heat exchanger is considered the most promising configuration as a latent heat storage system, because it provides a great digress of effectiveness for a minimum volume [23]. Ismail and Alves [24] made a theoretical model of the shell and tube-type heat exchanger for energy storage. A similar problem was modeled by Cao et al. [25]. In their models, the process of recovery and charging is carried out by the heat charging and recovery processes. The shell wall of the storage unit was considered adiabatic for both of these models. Bellecci and Conti [22] also investigated a model of energy storage in a shell and tube-type heat exchanger and used the enthalpy model to solve the problem. The latent heat energy storage systems were separately examined for counter and annular flows. It was recognized that the counter flow flowed storage system is more effective in absorbing thermal energy [23, 26]. Compared to the vertical tube-in-shell storage geometries, few surveys have been conducted on the horizontal ones. Increasing heat transfer by using finned surfaces is one of the techniques used for increasing the amount of energy storage. A great number of investigations, both experimental and theoretical, have been performed to explore the impact of rectangular cross-sectioned fins on the melting and solidification rate. For example, Bathelt and Viskanta [27] conducted a study of the solidification on tubes with horizontal fins placed at four various fin spacing distances. Cabeza et al. [28] also conducted different studies on the effect of various types of finned-tubes on frozen layers.

The presented literature survey suggests that compared to the vertical tube-in-shell storage geometries, a few investigations have been conducted on the horizontal ones. In particular, the heat transfer enhancement of PCMs in shell and finned-tube heat exchanger is still not entirely understood. The objective of the present research was to experimentally examine paraffin's melting and solidification attributes in a horizontal finned shell and tube storage geometry to increase solar thermal energy saving time. The storage geometry is on annular space (in which PCM is loaded) between a shell (a finned shell and tube-type of heat exchanger system) and a tube through which HTF (water) flows. In order to investigate the impact of the HTF inlet flow on the amount of charge, several experiments were carried out. In line with the focus of this survey, close attention was particularly paid to understanding the physics of the melting and solidification attributes based on the time records and histories of temperature patterns within the PMC; this served as a foundation for enhanced design and performance.

## 2. Experimental Details

### 2.1. Heat Storage Material

Paraffin ASTM D 87, which is provided from the Sigma-Aldrich, was employed as PCM in this study. Paraffin is widely recognized as a nontoxic, chemically stable, and attractive material which is degrade-less and possesses a notable capacity of storing latent heat within a narrow temperature range.  Table 1 presents a list of thermophysical properties which are employed in this research.

### 2.2. Experimental Apparatus and Procedure

**Figure 1:**
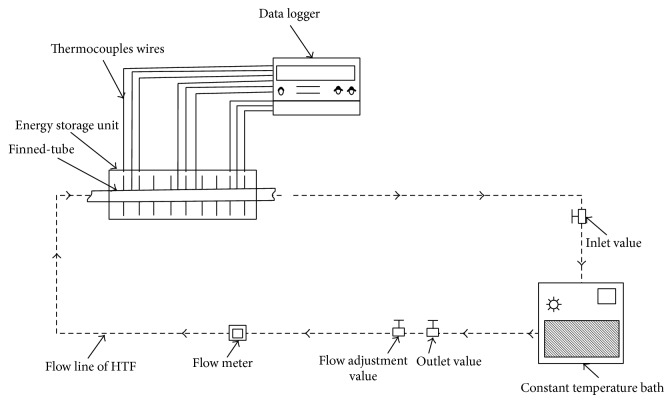
Schematic view of the experimental setup.

The experimental installation shown in Figure 1 is composed of heating/cooling circuit. The setup consists of a data acquisition unit (Computer and Data logger), connecting pipes, a horizontal test module (PMC storage container), a flow-adjustment valve, an outlet valve, and a constant temperature bath. The constant temperature bath (Wise Circu WCR-P6), which controls the heat transfer fluid's (HTF's) inlet temperature which delivers as much as 15 L/min of water, has an accuracy of ±0.1°C, and its temperature range is −20 to +100°C. The HTF utilized was distilled water. The constant-temperature bath of the circuit is a hot water in a finned-tube where the water is heated to the desired working temperature controlled thermally within ±1°C. This thermal bath served as a heat source replacing solar heat in real application.

In charging process, the heat water withdrawn by the constant-temperature bath passes through the flow meter in the form of a calibrated orifice plate and then flows through the test finned-tube fixed horizontally in the acrylic shell full with PCM (paraffin). This process was deemed to have finished once all temperature recordings of the test module (PCM container) showed higher values than PCM's melting temperature and the entire paraffin melted. This cycle was repeated subsequent to the charging process, and the energy stored was extracted through the discharging process (the circulation of cold water). At solidification process fluid at a much lower temperature than the liquid PCM which passes in the finned-tube heat is withdrawn from the liquid PCM and a layer of solidified PCM is formed over the tube and fins surfaces. The water then flows back to constant-temperature bath where it is further cooled and repumped to the test section. The solidification process stopped when the whole amount of PCM was solidified and the test module temperature was less than PCM's solidification temperature. The test module is composed of a copper tube with 15 mm inner and 20 mm outer diameters, which is surrounded by a shell tube with 60 mm inner and 64 mm outer diameters that provides an annular gap around the tube (tube-in-shell). The shell is made of acrylic with thermal conductivity (*k* = 0.1 W/m K). The PCM heads to fill up the test module of the 0.8 kg shell.

**Figure 2:**
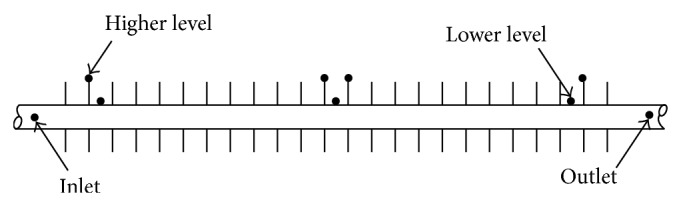
Installation of the thermocouples on the finned-tube.

The temperature measurement system consists of thermocouples and a data logger. It is essential to measure the temperatures of the finned-tube wall, the fin tip, and base tube to accurately determine the heat transfer to the phase-change material. In this study, *K*-type thermocouples were used, whose locations are depicted in Figure 2. During this experiment, the temperatures at 20 different points in the energy storage unit were measured and recorded. To enhance experiment accuracy and collect additional temperature data, many more thermocouples were applied among the fins. The thermocouples were located on three parts of the device. Sixteen thermocouples were used on the finned-tube. Two thermocouples were installed at entry and exit of the finned-tube to measure the temperature of the working fluid and another two thermocouples were utilized to measure the ambient temperatures and PCM container. The output data was recorded by the help of a HP type 34970A data logger with ±1°C accuracy; this data logger measured the thermocouples' millivolt outputs. The signal output from the data logger was transferred and documented in a personal computer.

For this process, HP-Benchlink software was used. The temperature values were scanned by the data logger every 10 seconds during both melting and solidification. All thermocouples were installed on the finned-tube by brazing. The HTF's volumetric flow rate was controlled by controlling the adjustment valve gate that was situated at the inlet of the loop. All experiments were conducted in a conditional room with ambient temperature of 30°C. The experiments were carried at no less than three times, and three different Reynolds number values of heat transfer fluid with similar outcomes were considered, namely, 1000, 1500, and 2000. The entire recorded transient temperatures were repeated in a range of ±3%. In an attempt to put a stop to loss into the vicinity, the system was covered with fiberglass insulation that was 37 mm thick and had an aluminized outer surface cladding. The average thermal conductivity of this cover layer was 0.038 W/m K. It is important to remember that studies on charging and discharging were both conducted at a constant inlet temperature. The discharging studies were carried out after the charging studies were conducted and were performed at Ti = 63 and 47°C. In section three, the studies on charging and discharging outcomes are separately presented. All instruments used in the test were calibrated prior to measurements. The measurements and photographs were taken in the region of the all fins where a precision scale is attached.

## 3. Numerical Procedure

The FLUENT commercial code based on finite volume method, which has been used in some previous works [29–31], was applied to solve the Reynolds Averaged Navier-Stokes (RANS) equations. This method is based on a particular type of the residual weighting approach. In this approach, the computational zone is divided into finite control volumes as each node is covered by a control volume. Eventually, the differential equation is integrated on each finite volume [32, 33]. The second-order upwind method [34, 35] was chosen for the discretization of all terms, while the SIMPLE algorithm [36, 37] was employed for pressure-velocity coupling. The solution was converged when the residuals for all the equations dropped below 10^−6^ [38, 39].

## 4. Results and Discussion

### 4.1. Charging Process

**Figure 3:**
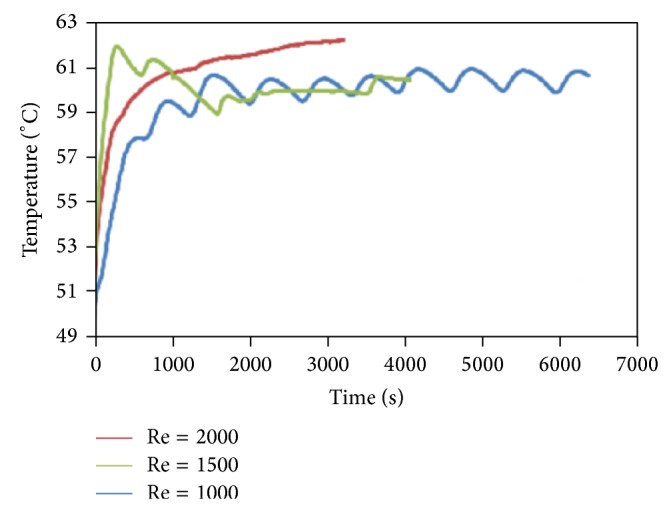
Temperature measurement records in the melting process for Re = 1000, Re = 1500, and Re = 2000.

Figure 3 shows the temperature gradient of PCM at different times and Reynolds numbers, Re = 1000, 1500, and 2000. It can be seen that PCM takes a short time to reach the melting temperature with a higher Reynolds number. According to results, the gradient temperature fluctuations began from 470, 276, and 270 seconds for Re = 1000, Re = 1500, and Re = 2000, respectively. It is clear that the fluctuation of gradient temperature decreased and became smooth with increasing Reynolds number. As a result, by increasing the Reynolds number in the charging process, the theoretical efficiency rises. As theoretical efficiency approaches unity (e.g., in the melting process), from the definition of efficiency, it can be concluded that the energy required to melt the whole PCM is provided [40].

**Figure 4:**
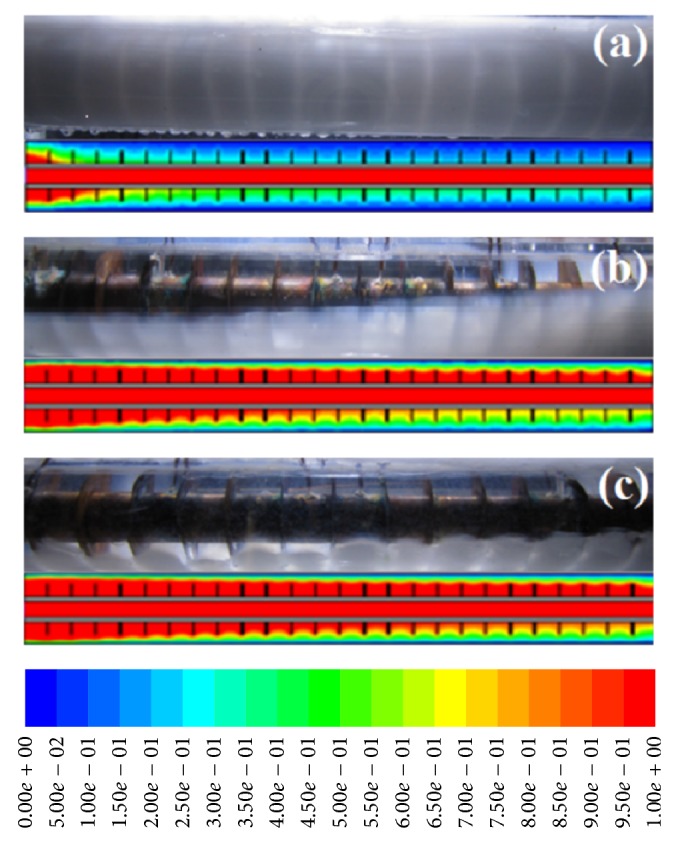
Melting process for Re = 1000 at (a) 1000 s, (b) 3500 s, and (c) 6000 s.

For a clearer explanation, Figures 4(a)–4(c) depict the results of Re = 1000 as an example of normal behavior of a transition. As witnessed, melting begins peripherally, near the HTF tube's wall and it continues to spread radially outwards. When the charging process is initiated, the PCMs close to the HTF tube's wall surface reach the melting point, which is due to the conduction of the finned-tube. The rapid melting of the PCMs near the walls is mostly because of greater temperature differences of PCM in the surrounding areas of the tube wall. Nevertheless, it is worth noting that the melting behavior of PCMs in the upper areas differs from those in lower regions. The molten PCM moves up towards the storage container's top areas due to the natural convection. The melting region extends radially upward. As a result, the areas in the upper sections attain melting temperature more rapidly than the lower parts.

The natural convection has a greater effect in the upper regions of the annular storage container. Aydin et al. [41] offered a good, comprehensive description of the physical attributes displayed by PCM in the charging process whereby two areas coexist. These two areas are the solid phase nonmelted PCM and liquid phase melted PCM. Due to buoyancy forces produced by the density gradients that resulted from differences in temperature, the melted PCM recirculation is spurred by the heat transfer through convection when the PCM's solid matrix melts. The heating and mixing of the molten PCM are elevated by recirculation inside the test section and the fact is that it takes less time for the areas closer to the upper regions to reach melting temperature than lower regions. However, it is worth noting that PCM has a lower density in the molten phase compared to the solid phase. In cases with larger operation, the PCM molten area spreads to cover larger areas of the PCM container. In line with the assumptions, the melting will increase with rising Reynolds number.

### 4.2. Discharging Process

**Figure 5:**
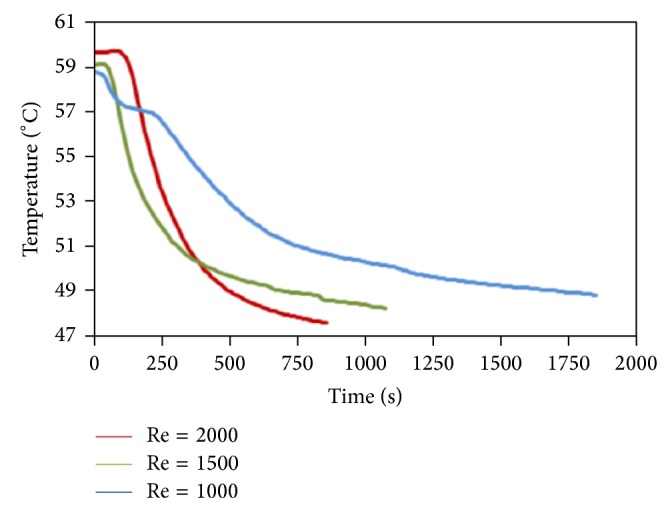
Temperature measurement records in the solidification process for Re = 1000, Re = 1500, and Re = 2000.

**Figure 6:**
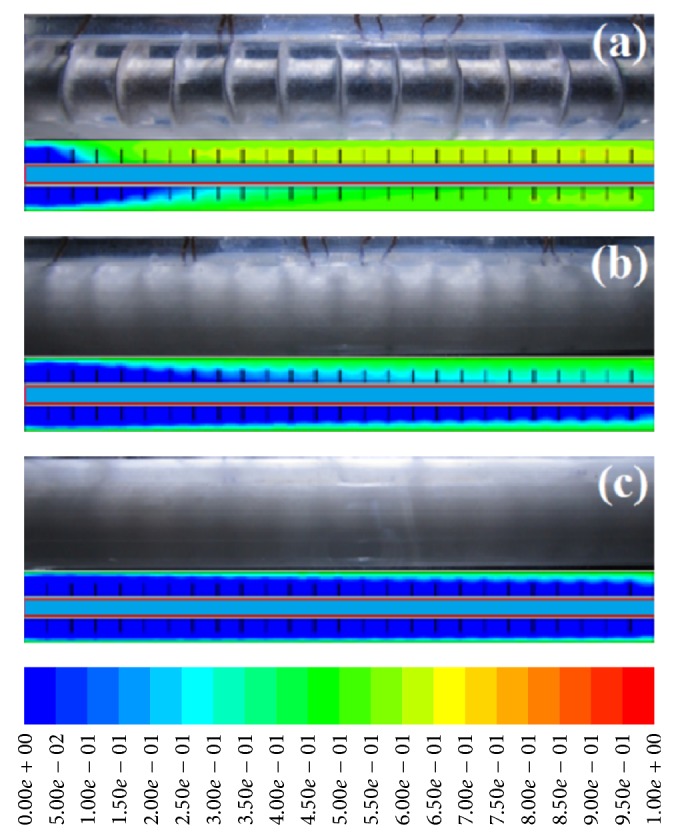
Solidification process for Re = 1000 at (a) 200 s, (b) 1000 s, and (c) 1800 s.

Experimentations on the discharging process were carried out by reversing the heat transfer direction between HTF and PCM. Hereby, the cold water was circulated through the system. The temperature measurement records for different thermocouples at varying times are presented in Figure 5. The initial temperature of PCM, in the liquid phase, is 63°C. It is then introduced to a sudden temperature change in the circulating system during the discharging process as a result of the high temperature differences between HTF and PCM. Due to the transfer of sensible heat taking place within the solid PCM, volume increases until complete solidification occurs at solidification temperature. In accordance with the expectations, the points closer to the inlet part and HTF wall solidified faster than the other areas. In an attempt to provide a more comprehensive perception of the physics of the process, Figure 5 presents the temperature distribution's temporal variation at the lengthy points that are similarly distanced from the tube wall of HTF. The PCM temperature at particular positions reduces slowly with time, as demonstrated before; temporal variation becomes almost uniform after some time due to the initial impact and effectiveness of natural convection on heat transfer. After that, conduction becomes the only dominating mechanism in heat transfer, with lower convection impacts in contrast to the melting or charging instances. This happens due to the melted PCM's circulation quantity that is influenced by the decreasing natural convection with the increase in time as a result of the outward HTF tube's solidification. In the same vein, the process of solidification will speed up with increasing Reynolds number in the discharging process. For a clearer explanation, Figures 6(a), 6(b), and 6(c) depict the results for Re = 1000 where the ordinary and normal behavior of a transmission occurs. It is clear that solidification occurred homogeneously with increasing time.

## 5. Conclusion

An experimental investigation of the melting and solidification behavior of paraffin is presented in the current study. A thermal unit of storing energy, which has a horizontal finned-tube heat exchanger, was selected. According to the experimental results, the melting behavior of the points situated in the upper areas is drastically different from those in lower areas; moreover, this behavior repeated from the inlet points to the exit points around the finned-tube. The experimental results also confirmed that the natural convection currents lead to the upward movement of molten PCM to the storage unit's upper regions. An asymmetric temperature field is created due to the radially upward extension of the melting area. For this reason the points in the upper left areas reach melting temperature faster than the ones in the lower right areas. By examining the solidification behavior, it was identified that the natural convection is initially effectual on heat transfer, which is later surpassed by conduction. As expected, increasing the Reynolds number is believed to improve the process of phase change within the PCM. The heat transfer rate and consequently the time to complete the melting process are directly related to Reynolds number. The present study demonstrated that, by increasing the Reynolds number from 1000 to 2000, the total melting time decreases by 58%. It should be noted that decreasing charging time and increasing discharge time are important subjects that should be investigated more in future work. This perceived asymmetric behavior may lead to the creation of new, innovative designs.

## Figures and Tables

**Table 1:** Thermophysical properties of the PCM and finned-tube.

Properties	Tube material	Phase change material	
		Solid	Liquid
*ρ* (kg/m^3^)	8800	0.790	0.916
*C*_*p*_ (j/kg·K)	420	n.a	n.a
*K* (W/m·K)	52	0.167	0.346
*α* (m^2^/s)	1.4 × 10^−5^	n.a	
*L* (kj/kg)	n.a	160	
*T* (°C)	n.a	53	57
